# Glial cell biology in the Great Lakes region

**DOI:** 10.1186/s12974-016-0534-6

**Published:** 2016-03-31

**Authors:** Douglas L. Feinstein, Robert P. Skoff

**Affiliations:** Department Anesthesiology, University of Illinois-Chicago, Chicago, IL 60612 USA; Jesse Brown VA Medical Center, Chicago, IL 60612 USA; Department Cell Biology and Anatomy, Wayne State University, Detroit, MI 48202 USA

**Keywords:** Astrocyte, Microglia, Schwann cell, iPSC, Stem cell, Aging

## Abstract

We report on the tenth bi-annual Great Lakes Glial meeting, held in Traverse City, Michigan, USA, September 27–29 2015. The GLG meeting is a small conference that focuses on current research in glial cell biology. The array of functions that glial cells (astrocytes, microglia, oligodendrocytes, Schwann cells) play in health and disease is constantly increasing. Despite this diversity, GLG meetings bring together scientists with common interests, leading to a better understanding of these cells. This year’s meeting included two keynote speakers who presented talks on the regulation of CNS myelination and the consequences of stress on Schwann cell biology. Twenty-two other talks were presented along with two poster sessions. Sessions covered recent findings in the areas of microglial and astrocyte activation; age-dependent changes to glial cells, Schwann cell development and pathology, and the role of stem cells in glioma and neural regeneration.

## Schwann cells in health and disease

The contributions of Schwann cells (SC) to peripheral neuropathy were covered in several talks. Larry Wrabetz (University of Buffalo, Buffalo, NY) started off the meeting with a brief review of possible causes of hereditary peripheral neuropathies, citing Charcot-Marie-Tooth 1B (CMT-1B) disease as an example due to a deletion in the myelin P0 protein. Surprisingly, the mechanism by which this mutation induces pathology is not directly related to its role in myelination, but instead to induction of the unfolded protein response (UPR). On that basis, Wrabetz carried out studies in mice harboring the P0 deletion using Sephin1, a new holophosphatase inhibitor. Sephin1 selectively binds to and inhibits the stress-induced phosphatase PPP1R15A, reduced the consequences of the UPR, and prevented dysmyelination and motor function pathology. The findings have a potentially large impact, since endoplasmic reticulum stress, the UPR, and pathological proteostasis have been documented not only in patients with CMT, but also in neurodegenerative diseases such as Alzheimer’s Disease. This suggests that pathological proteostasis may represent a common therapeutic target for a variety of neurodegenerative diseases.

Jun Li (Vanderbilt University, Nashville, TN) organized a session to discuss molecular signals underlying Schwann cell (SC) maturation and myelination activities. One class of molecules gaining increased interest is extracellular matrix (ECM) molecules. Recent studies presented by Kelly Monk (Washington University, St Louis, MO), show that a class of G-protein coupled receptors termed adhesion GPCRs (aGPCRs), are major regulators of SC development and myelination. The aGPCRs are 7-transmembrane domain-containing proteins with long extracellular N-termini. Within the terminus region are conserved domains that allow interactions with other proteins, as well as a GPCR auto-proteolysis-inducing (GAIN) domain and a GPCR proteolytic site (GPS). Upon activation, cleavage occurs leading to release of the N-terminus and induction of intracellular signaling pathways. Monk went on to show that in zebrafish, the aGPCR GPR126 is critical for radial sorting of axons and myelination. Understanding the activities and regulation of GPR126 (and other related aGPCRs) may therefore provide a means to stimulate remyelination following injury or during disease.

Proteolytic cleavage appears to play many important roles in SC maturation and function. Riqiang Yan (Cleveland Clinic, Cleveland, Ohio) presented data showing that the BACE1 (B-site APP cleaving enzyme 1, which has important roles in the non-amyloidogenic processing of APP) is needed during development for proper myelination, and in adults for remyelination of injured sciatic nerves. However, the targets of BACE1 within SCs are not clear. Yan presented data indicating that cleavage of neuregulin type 1 (Nrg1) within SC is critical to the ability of the cells to remyelinate injured nerves. Considering what is already known regarding BACE1 in the context of AD, these findings open up new possibilities for increasing peripheral remyelination by selectively activating BACE1 in SCs.

Inherited mutations in the PMP22 gene are associated with the most common types of inherited neuropathies, including hereditary neuropathy with liability to pressure palsies (HNPP) caused by PMP22 deficiency. Interestingly, PMP22 deficiency causes impaired propagation of nerve action potentials in the absence of demyelination, suggesting other functions for PMP22. Jun Li showed using Pmp22+/− mice as a model of HNPP the presence of disruptions in multiple types of cell junction complexes in peripheral nerve, associated with increased myelin permeability and impaired action potential propagation. Since PMP22 is known to interact with immunoglobulin domain-containing proteins such as junctional adhesion molecule-C (JAM-C) and myelin-associated glycoprotein (MAG), he described studies to show that deletion of *Jam-c* or *Mag* recapitulates the neuropathology observed in HNPP. These studies reveal a novel mechanism by which PMP22 deficiency can affect nerve conduction which does not involve myelin removal but instead disruption of myelin junctions.

## Novel regulators of microglial function

The importance of microglial cells in virtually all types of neurological conditions and diseases is now well-accepted, requiring an understanding of their protective roles as well as their pathological roles. Jyoti Watters (University of Wisconsin, Madison, WI) organized a session addressing studies to determine how microglial functions are regulated at the epigenetic level, to identify similarities of actions in diverse disorders (migraine and multiple sclerosis), and to understand how aging alters their functions.

Epigenetic modifications occur following various stimuli, including inflammatory activation. Watters described studies of epigenetic modifications in microglia during periods of chronic neuroinflammation induced by repetitive intermittent hypoxia, a model of sleep apnea. It was known prior to these studies that in this model, the microglial phenotype shifts to a pro-inflammatory state (M1) early during pathology, and then to a reparative or neurosupportive state (M2) at later times. She showed that in this model, the activities of the Jumonji family of histone demethylases were necessary for the M1 phenotype, while expression of miRNAs appeared important for dampening the inflammatory response and enabling an M2 phenotype. Surprisingly, the activity of toll-like receptor 4 (TLR4) was necessary for transition to both the M1 and M2 phenotypes, pointing to a dual role for this key molecule. It is suggested that similar regulatory mechanisms may be involved in microglial adaptation to situations of chronic neuroinflammation in other models of neural injury and degeneration as well.

Richard Kraig (University of Chicago, Chicago, IL) presented work which highlighted similarities between migraine and multiple sclerosis (MS), two seemingly disparate diseases. Migraine occurs two to three times more frequently in MS patients, and patients suffering from migraine with aura display white matter abnormalities. He presented in vitro and in vivo data showing that spreading depression (SD), the underlying cause of migraine with aura, produced abnormalities in myelin mediated by immune cell activation and IFNγ production, similar to MS. Importantly, IFNγ appears to have a dual role in these diseases, since environmental enrichment (EE), intranasal IFNγ delivery in animals, or phasic application of IFNγ in slice cultures reduced oxidative stress, increased myelin proteins, and increased the threshold for SD. How these protective actions of IFNγ occur is unclear, but Kraig posited that exosomes released following EE or from IFNγ-stimulated microglia contain miRNAs that reduce oxidative stress and promote myelination. The work suggests that IFNγ, in the absence of other factors that normally trigger immune activation and exacerbates disease, can instead promote the production of exosomes that enhance brain health.

## The role of astrocytes in health and disease

Accumulating evidence indicates that astrocytes and neuroinflammation can either be detrimental or beneficial in neurological diseases. Sandra Hewett (Syracuse University, Syracuse, NY) organized a session to discuss the pros and cons of astrocyte immunomodulatory signaling in CNS disease. The role of astrocyte TGFβ signaling in toxoplasmic encephalitis―an inflammatory disease of the CNS caused by infection with Toxoplasma gondii―was explored by Marion Buckwalter (Stanford University, Stanford, CA). Using a mouse model of infection, Buckwalter showed that, despite no differences in Toxoplasma burden, mice with impaired astrocytic TGFβ signaling exhibited twofold greater neuronal damage and increased myeloid cell activation and lymphocyte infiltration into the CNS compared to controls. The data suggest that astrocyte TGFβ limits peripheral immune infiltration and injury after infection. As TGFβ upregulation occurs in other acute brain injuries and diseases, TGFβ signaling may present a universal pathway by which astrocytes limit neuroinflammation and injury.

M. Kerry O’Banion (University of Rochester, Rochester, NY) demonstrated previously that sustained astrocytic IL-1β expression reduced amyloid beta (Aβ) plaque size and frequency in a mouse model of AD. O’Banion now reported that persistent expression of IL-1β in brains of those mice led to an IL-4-dependent alternative activation of microglia that played an essential role in Aβ clearance. By contrast, using an antibody to block IL-4 signaling, he showed a reduction in arginase-1-positive microglia in IL-1b-expressing mice, coincident with reduced Aβ clearance. These data suggest that neuroinflammation-dependent changes in microglial phenotype impact Aβ plaque load, and potentially other neuropathologies.

Continuing the study of IL-1β, Hewett described its paradoxical role in acute CNS injury and protection. Previous work from her group demonstrated that injury facilitated by IL-1β upregulation in stroke was likely mediated by enhancement in astrocyte system X_c_^−^ activity. However, she presented data detailing that this enhancement could increase also astrocyte glutathione (GSH) production, thereby reducing susceptibility to oxidant injury. IL-1β reduced production of reactive oxygen species in astrocytes generated by exposure to tert-butyl hydroperoxide, and that toxicity (or that due to iron) was attenuated by IL-1β. Protection was reversed by concomitant exposure to L-buthionine-S,R-sulfoximine, which prevented the IL-1β-mediated rise in GSH production. Given these results, Hewett posited that the increase in IL-1β expression after stroke insult or injury may be part of a protective response which ultimately goes awry.

Finally, John Bethea (Drexel University, Philadelphia, PA) described the role of astroglial NF-κB signaling in models of MS. Extending earlier findings that inactivation of astroglial NF-κB in vivo improved functional outcome in experimental autoimmune encephalomyelitis (EAE), Bethea presented evidence showing that the key element driving recovery was reduced immune cell infiltration into the CNS at the chronic phase of disease, occurring independently from alterations in blood brain barrier permeability. Further, he presented results demonstrating a reduction in the number and activation state of resident microglia, and in the Th1 and Th17 profile of infiltrating T-cells during acute disease. Collectively, these data underscore that astrocytes, either directly or indirectly, play a critical function in regulating CNS inflammation that follows EAE, hence driving disease progression and severity.

## The aging glial cell

It was clear from several presentations that microglial function is regulated by environmentally induced epigenetic changes, and to production of exosomes which contain miRNAs, another form of epigenetic reprogramming. Similar events occur in most, if not all glial cell types. Patrizia Casaccia (Icahn School of Medicine at Mount Sinai, New York, NY) described studies to define changes in the “epigenetic landscape” of oligodendrocytes during aging. It was already known that oligodendrocyte maturation is accompanied by specific histone modifications including decreased acetylation and increased trimethylation of histone H3-K9 and K27. She showed that aging reverses those changes, and is associated with re-expression of transcriptional inhibitors as well as inefficient myelin repair in older mice. She then presented new data showing that molecules which block interactions of acetylated lysines with bromodomain-specific proteins allow oligodendrocytes to overcome the blocks induced by hyperacetylation. The potential of epigenome modulators therefore could include reversal of age-related epigenetic dysfunction in oligodendrocytes.

The ways that microglial functions change with age is critical to understanding and developing interventions for neurodegenerative diseases for which age is the greatest risk factor. Anne Boullerne (University of Illinois, Chicago, IL) organized a session to address the consequences of aging on glial cell function. Diana Norden (Ohio State University, Columbus, OH) described how microglial inflammatory activities shift during aging to become “primed” or more highly pro-inflammatory. The aged microglia have an amplified and prolonged pro-inflammatory response that results from impairments in key anti-inflammatory systems coming from astrocytes. She reported that aged astrocytes have reductions in IL-10R1 expression, which causes the astrocytes to host aberrant responses to IL-10. This results in reduced TGFβ signaling to the microglia, allowing for increased inflammatory responses. Thus, understanding how aging changes normal astrocyte-microglia communication is necessary to identify novel targets for therapeutic interventions.

Wolfgang Streit (University of Florida, Gainesville, FL) presented images from human autopsy samples of controls and AD patients to understand the changes that occur to microglia cytoplasmic structure as a result of cell senescence. Streit hypothesized that Alzheimer-type neurofibrillary degeneration (tau pathology) occurs as a consequence of an aging-related decline in microglial ability to support neurons. He showed that the appearance of tau-positive structures (neurofibrillary tangles, neuropil threads, and senile plaques) in the entorhinal cortex and surrounding temporal gyri is preceded and accompanied by widespread microglial dystrophy. The extent of microglial degeneration correlates positively with development of neurodegeneration in both AD and Down syndrome, supporting the hypothesis of microglial dysfunction. At the same time, he was unable to detect the presence of activated microglia in the human temporal cortex, providing evidence against the longstanding idea that neuronal degeneration is a result of overly activated, out-of-control microglia. Instead, he believes that the brain’s innate immune system is subject to an aging-related decline in function due to the senescent deterioration of microglia. The implications for potential therapeutic approaches are profound: instead of trying to suppress microglia with anti-inflammatory drugs to prevent neurodegeneration, it may be more effective to develop agents that can stimulate microglial activity.

The effects of aging on glial cells can be further influenced by gender; which could help account for gender differences in the incidence of neurological conditions and disease such as MS, AD, and stroke. Farida Sohrabji (Texas A&M Health Science Center, Bryan, TX) presented data regarding changes in astrocytes that occur during aging and during recovery from ischemic stroke. Not only do women suffer more severe stroke than men, but this phenomenon is replicated in animal studies where aging females have more extensive brain loss compared to younger females. Sohrabji showed that, in comparison to young females, astrocytes from aged female rats secrete less IGF-1 (which shows beneficial effects in stroke), have reduced capacity for glutamate clearance, and paradoxically synthesize higher levels of inflammatory cytokines. In co-cultures, aging astrocytes showed a greater propensity to recruit monocytes across a Transwell system, indicating a potential for a more severe inflammatory response. Sohrabji showed that the age-dependent changes in astrocytes involve epigenetic modifications, with astrocytes from the ischemic cortex of adult female rats having more histone H3 lysine 4 trimethylation than middle-aged female astrocytes. Collectively, these studies show that the functional capacity of aging astrocytes is impaired and may be associated with the greater stroke impairment seen in aging females.

Is there a way to combat the consequences of aging in glial cells? Carmela Abraham *(*Boston University School of Medicine, Boston, MA) discussed the roles that the anti-aging protein Klotho has in the early differentiation of oligodendrocytes and their capacity for remyelination in the adult CNS. Klotho, which is significantly downregulated in the aged brain, was discovered to play a role in aging when Klotho-deficient mice were found to exhibit signs of aging and premature death. Abraham showed that Klotho-knockout mice suffer from severe hypomyelination. Furthermore, the addition of Klotho to oligodendrocyte progenitor cells induced their maturation to myelin-producing oligodendrocytes. Most interestingly, Klotho-overexpressing mice show better cognition in a number of behavioral tasks. Finally, when demyelination was induced by cuprizone, the number of remyelinated axons was twice as high in Klotho overexpressing mice compared to controls. These findings raise the prospect that methods to increase Klotho, or provide small molecular weight mimetics, could be used to improve remyelination as well as cognitive deficits in neurological disorders and diseases.

## The Janus face of stem cells: roles in neuroregeneration and cancer

Given the age and disease-related changes in glial cells that likely worsen neuropathology, one must consider methods to replace or correct those deficiencies. Towards those goals, Margot Mayer Proschel (University of Rochester, Rochester, NY) organized a session to discuss the potential use of stem cells as therapeutics, and how knowledge of stem cell biology can inform us of mechanisms that underlie diseases.

The potential of astrocyte-based therapies for CNS repair was presented by Chris Proschel (University of Rochester, Rochester, NY). He shared data that demonstrated the ability of astrocyte transplants to promote motor recovery in rat models of both traumatic spinal cord injury and in a neurotoxic model of PD. His work underscored the versatility of astrocytes to restore neuronal function of the host even after delayed transplantation. Importantly, the therapeutic benefits were only observed with a specific subpopulation of astrocytes generated in vitro by directed differentiation from stage-specific precursor populations using bone morphogenetic protein-4. Proschel’s work demonstrated that the therapeutic potential was associated with distinct expression patterns of growth factors, extracellular matrix proteins, and synaptogenic factors, thus highlighting the importance of using the appropriate precursor population and differentiation method to obtain therapeutically competent astrocytes.

Mark Noble (University of Rochester, Rochester, NY) extended the discussion of therapeutic strategies and presented an integration of progenitor cell biology, redox biology, signaling pathway analysis, and drug discovery that is providing new therapeutic approaches for glioblastoma (GBM) treatment. Analyses of glial progenitor cell function had previously revealed that small changes in intracellular redox state are critical in modulating survival and differentiation. The central mechanism controlling these outcomes appears to be the redox/Fyn/c-Cbl pathway. Activation of this pathway promotes lysosomal degradation of important receptor tyrosine kinases including PDGRα and EGFR, with subsequent suppression of downstream signaling. c-Cbl can be inhibited upon complex formation with a guanine nucleotide exchange factor called Cool-1/ß-pix. Noble reported that c-Cbl activation may be essential for GBM tumorigenesis, since Cool-1/ß-pix knockdown eliminated the ability of GBM cells to generate tumors, increased their sensitivity to chemotherapeutic agents, and restored the ability to activate c-Cbl. Noble highlighted the tumor selective nature of this pathway as Cool-1 knockdown in primary glial progenitor cells did not have these effects. Based on these findings, Nobel reported discovery of several drugs, previously approved for other purposes, that restore c-Cbl function in GBM cells. These drugs confer sensitivity to standard of care treatments for GBM both in vitro and in vivo, and provide exciting opportunities for moving rapidly to clinical trials.

Biplab Dasgupta (University of Cincinnati, Cincinnati, OH) further expanded upon the role of metabolic changes in GBMs, and discussed new studies focusing on the liver kinase B1 (LKB1):adenosine monophosphate kinase (AMPK) pathway which regulates cellular metabolic processes. Dasgupta found several AMPK subunits and active AMPK overexpressed in human GBMs and in mouse models of high-grade glioma. Silencing AMPK reduced the viability of patient-derived primary GBM cells in vitro and suppressed tumor growth in vivo. His work suggests that in the context of GBMs, AMPK positively regulates the transcription program of both glycolysis and mitochondrial respiration and thus influences the global bioenergetic program of GBM.

The role of LKB1 in CNS was further discussed by David Braun (University of Illinois, Chicago, IL). He presented data showing that loss of LKB1 from primary cortical astrocytes was associated with an increase in proliferation, a loss of contact inhibition, induction of anchorage-independent proliferation, and enhanced migratory capability; all properties similar to those observed in GBMs. He went on to show that the LKB1 null astrocytes expressed known markers of pluripotency including Sox2, Oct4, and Nanog, suggesting that lack of LKB1 caused de-differentiation of the astrocytes towards a more primitive cell type. He then showed tantalizing data that these “stem-like” cells could be induced to differentiate into oligodendrocytes or neurons in vitro depending upon growth conditions. Braun suggested that inhibition of LKB1 activity may represent a novel way of reprogramming astrocytes, which could help improve efficiency of reprogramming of other cell types, or of developing new models of GBMs.

## Conclusions

Robert Skoff (Wayne State University, Detroit, MI) summarized the presentations and highlighted the contributions of the younger investigators who constituted roughly 50 % of the attendees. As discoveries in glial cell biology continue to emerge, all look forward to the next meeting to take place in 2017.

## Abbreviations

AMPK: adenosine monophosphate kinase
APP: amyloid precursor protein
Aβ: amyloid beta
CMT: Charcot-Marie-Tooth
EAE: experimental autoimmune encephalomyelitis
ECM: extracellular matrix
EE: environmental enrichment
GBM: glioblastoma
GPCR: G-protein coupled receptor
GSH: glutathione
iPSC: inducible pluripotent stem cell
LKB1: liver kinase B1
MS: multiple sclerosis
SC: Schwann cell
SD: spreading depression
TLR4: toll-like receptor 4
UPR: unfolder protein response
VEGF: vascular endothelial growth factor

